# Engineering Iron Oxide Nanoparticles for Clinical Settings

**DOI:** 10.5772/58841

**Published:** 2014-01-01

**Authors:** Aitziber L. Cortajarena, Daniel Ortega, Sandra M. Ocampo, Alberto Gonzalez-García, Pierre Couleaud, Rodolfo Miranda, Cristobal Belda-Iniesta, Angel Ayuso-Sacido

**Affiliations:** 1 Instituto Madrileño de Estudios Avanzados IMDEA-Nanociencia, Madrid, Spain; 2 Centro Nacional de Biotecnología (CNB-CSIC) - IMDEA Nanociencia Associated Unit “Unidad de Nanobiotecnología”, Cantoblanco, Madrid, Spain; 3 Institute of Biomedical Engineering, University College London, UK; 4 Centro Integral Oncológico Clara Campal (CIOCC) and Instituto de Medicina Molecular Aplicada (IMMA). Hospital de Madrid Foundation, Madrid, Spain; 5 National School of Health, ISCIII, Madrid, Spain

**Keywords:** Iron Oxide Nanoparticles, IONP, SPION, USPION, VSPION, Drug Delivery, Magnetic Hyperthermia, MRI, Nanomedicine

## Abstract

Iron oxide nanoparticles (IONPs) occupy a privileged position among magnetic nanomaterials with potential applications in medicine and biology. They have been widely used in preclinical experiments for imaging contrast enhancement, magnetic resonance, immunoassays, cell tracking, tissue repair, magnetic hyperthermia and drug delivery. Despite these promising results, their successful translation into a clinical setting is strongly dependent upon their physicochemical properties, toxicity and functionalization possibilities. Currently, IONPs-based medical applications are limited to the use of non-functionalized IONPs smaller than 100 nm, with overall narrow particle size distribution, so that the particles have uniform physical and chemical properties. However, the main entry of IONPs into the scene of medical application will surely arise from their functionalization possibilities that will provide them with the capacity to target specific cells within the body, and hence to play a role in the development of specific therapies. In this review, we offer an overview of their basic physicochemical design parameters, giving an account of the progress made in their functionalization and current clinical applications. We place special emphasis on past and present clinical trials.

## 1. Introduction

Over the last decade, nanotechnology has become more relevant in many industrial sectors, including medicine. In general, the future projection and promotion of iron oxide nanoparticles (IONPs) as a point of reference among other magnetic nanomaterials for clinical applications relies chiefly on their biocompatibility in moderate doses, their relatively well-known iron metabolic pathways and their ability to be produced in a wide range of sizes and shapes with biofunctionalization potential. Additionally, the added advantage of magnetic actuation makes IONPs stand out from many other nanotechnology-based therapeutic and diagnostic approaches. In any case, IONPs have already passed through the preclinical stage to become a reality in clinical practice. In particular, they improve imaging-based diagnostics of immunological diseases, cardiovascular and cerebrovascular pathologies and cancer. They show great promise to serve as a cell tracking system in cell-based therapies, and to generate local temperature increases in the magnetic thermotherapy of solid tumours.

The purpose of this work is to review the basic parameters involved in the design of IONPs and their functionalization possibilities which, in the next few years, will bring into being a completely new generation of IONPs with selective targeting properties. Functionalized and multifunctionalized IONPs will become nanocarriers that will improve the selectivity of specific cells and, therefore, allow for the development of specific therapies. We describe their biochemical features, their cell internalization and *in vivo* administration, as well as the reduced toxicology effects that have contributed to their successful use in current clinical applications. Additionally, we discuss most of the past and present clinical trials involving IONPs, placing special emphasis on their interactions with different compartments of the immune system. Finally, we highlight the potential advances in functionalization and magnetic hyperthermia as two fields that will surely push forward the clinical use of IONPs.

## 2. Basic physicochemical characteristics of IONPs

### 2.1 Size

This is arguably the most important parameter upon which any IONPs system must be designed. First, it profoundly affects the dynamics of the magnetic moments in magnetic NPs—also regarded as the magnetic *relaxation processes*—that also depend on other parameters, such as temperature or externally applied magnetic fields. Second, particle size is of paramount importance in the detection, internalization and eventual fate of IONPs inside mammalians.

Two of the most relevant magnetic scale lengths that characterize magnetic NP systems are the superparamagnetic radius (*RSPM*) and the single domain radius (*RSD*). *RSPM* refers to the maximum particle size up to which a superparamagnetic regime is observed. *RSD* indicates the value below which the formation of magnetic domains—regions grouping magnetic moments with the same orientation—is no longer energetically favourable and indicates that the most stable magnetic configuration for the particle is the single domain state [1]. Both *RSD* and *RSPM* can be worked out from the anisotropy constant (*K*), the dimensionless hardness parameter (κ) and the exchange stiffness constant (*A*) [2] of the material of interest. Table 1 summarizes these parameters for maghaemite and magnetite, the most commonly used iron oxides in biomedicine, and gives a glimpse of the range of magnetic behaviours of IONPs with varying particle size.

**Table 1.** Characteristic magnetic length scales for the most relevant iron oxides [123][124][125]. Data for iron are included for comparison.

	K	A	MS	k	lex	Rsd	Rspm
	(J/m3)	(J/m)	(A/m)		(nm)	(nm)	(nm)
Maghemite	4.6×10^3^	∼10^−11^	3.8×10^5^	0.16	7.4	42.5	17.5
Magnetite	1.35×10^4^	133×10^−11^	4.8×10^5^	0.21	6.8	52.7	12.2
Iron	4.8×10^4^	1.49×10^−11^	1.71×10^6^	0.11	2.0	8.3	8.0

IONPs may be produced by a number of methods that allow for precise control over their size, shape and surface chemistry [3][4][5][6]. These parameters, along with others, can be used to classify IONPs for different purposes [7]. With reference to particle size, the researchers working on IONPs in medicine and biology—especially in the MRI community—use an informal classification divided into distinct ranges as follows: below 10 nm, very small superparamagnetic iron oxide NPs (VSPIONs); between 10 and 50 nm, ultrasmall superparamagnetic iron oxide NPs (USPIONs); and between 50 and 180 nm, superparamagnetic iron oxide NPs (SPIONs).

Although this classification might be useful only for the purpose of gathering some size ranges showing similar circulation and relaxational properties for specific applications, IONPs are persistently referred to in the literature as SPIONs, regardless of their true magnetic properties. This misuse of the term SPION is both inaccurate and confusing, as the lower-end value of the SPIONs' size range lies at the theoretical transition between superparamagnetic and single domain states for iron oxides (Table 1). Thus, most of the particle sizes encompassed within this range would in fact be single domain, not superparamagnetic. Interparticle interactions and surface effects further complicate this picture by shifting *RSPM* away from its theoretical value. Therefore, we recommend avoiding, where possible, the SPION-based nomenclature and using a more general one describing the actual behaviour of the IONPs experimentally characterized by magnetic measurements, either: (i) based on size effects (superparamagnetic, single domain and multidomain) or (ii) on the coupling between magnetic moments (ferro-, ferri-, or antiferromagnetic, as applicable). Another source of confusion is the conflation of the numerical size values measured by different techniques, more specifically the “physical” size—typically obtained through electron microscopy, X-ray diffractometry, or similar means—and the *hydrodynamic size*—measured by dynamic light scattering or acoustic spectroscopy, and a value that, besides the physical particle size, also takes into account the thickness of any coating agent and the solvation molecules from the solvent. A final word of caution in this regard is that many IONPs are prepared by forming aggregates of variable size instead of a dispersion of single NPs; the size of the aggregates—often referred to as *multi-cores*—and that of the single particles are sometimes carelessly conflated.

**Figure 1.**
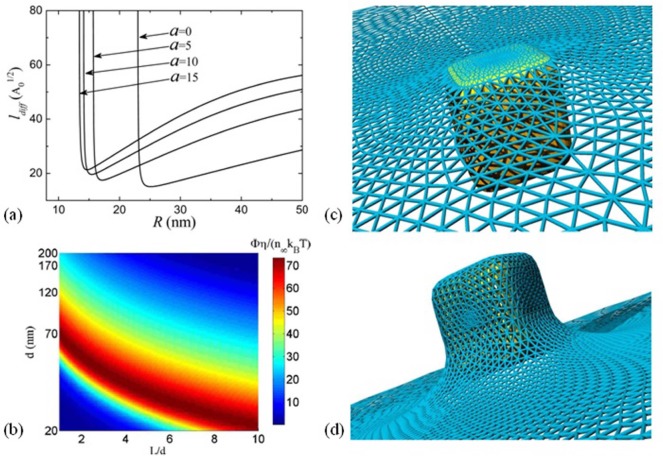
Membrane deformation for (a) shallow wrapping and (b) deep wrapping of a cubic-shaped nanoparticle. The network of edges and triangles describes the membrane shape and has been used for the numerical calculation of the curvature energy. [Adapted with permission from S. Dasgupta, *et al., Nano Letters* 14(2) (2014) 687–693. Copyright 2014 American Chemical Society]. (c) Values of diffusion length (*l_diff_*) of nanoparticles as a function of particle size (*R*) at fixed values of aspect ratio (*a*) [Reprinted with permission from X. Li, *Journal of Applied Physics*, vol. 111 (2012) 024702. Copyright 2012, AIP Publishing LLC]. (d) Normalized absorption rate of cylindrical particles with different diameters and aspect ratios [Permission pending].

From a biological point of view, IONPs can travel anywhere through the circulatory system of the human body, since the smallest capillaries are 4–6 μm wide [8]. IONPs under 100 nm are considered to be suitable for any application requiring tissue penetration, but those around 5 nm are more effective for tumour penetration [9]. Nonetheless, at the cellular level, NP size also influences the binding affinity of molecules, the uptake of additional particles into the cell, and the actual location within the cell. For a given NP shape, there is an optimal size for NP diffusion through the potential barrier characterizing the particle–cell interaction, as found by theoretical models [10][11]. Numerical calculations of these models indicate that the optimal NP size for a reasonably quick endocytosis is somewhere around 25 to 35 nm. Regardless of the particular considerations in each of the proposed models, the aforementioned optimum size range is given by the interplay between the kinetics and thermodynamics of the diffusion–absorption process of NPs relative to the thermal energy involved. Of particular note is that within the framework of the model proposed by Shi *et al.*, the optimum particle sizes obtained do not depend on the absorption coefficient, the viscosity of the medium, or the cell size [10] (Figure 1).

### 2.2 Shape

Consider a magnetic particle as composed of many positive and negative poles; these will cancel each other, but there will still be free poles at the surface of the particle. These free poles create a magnetic field—the demagnetizing field (*H_d_*)—that depends on the particle shape and is opposed to the direction of the external field (*H_ext_*). It follows that the effective magnetic field (*H_eff_*) acting on a particle will be different from *H_ext_*:*H_eff_ = H_ext_ -H_d_*, where *H_d_ = N·M*, with *M* the magnetization of the particle and *N* the so-called demagnetizing factor (*N*). In the case of a spherical shape, *N* = 1 due to the even distribution of free poles over the particle's surface. Other morphologies different from a perfect sphere—the usual case in many real systems—may be approximated to distorted ellipsoids. In this way, the anisotropy energy associated with the shape of an ellipsoidal particle with its major axis lying along the *z* direction equals [12]: *E_shape_* = [*μ0MS*^2^(*N_x_-N_z_)/*2]^−1^, where *MS* is the saturation magnetization and *μ0* is the permeability of the free space.

Besides its contribution to the total anisotropy energy of magnetic particles, the role of the particle shape in the magnetic properties of NPs is linked to the stability of the single domain configuration. For example, disc-like NPs with relatively higher aspect ratios may show a closed spiral arrangement of their magnetic moments [13], called a *vortex* state, whereas particles with the same shape but lower aspect ratios show the typical parallel arrangement of moments commonly seen in uniformly magnetized single domain NPs [14].

**Figure 2.**
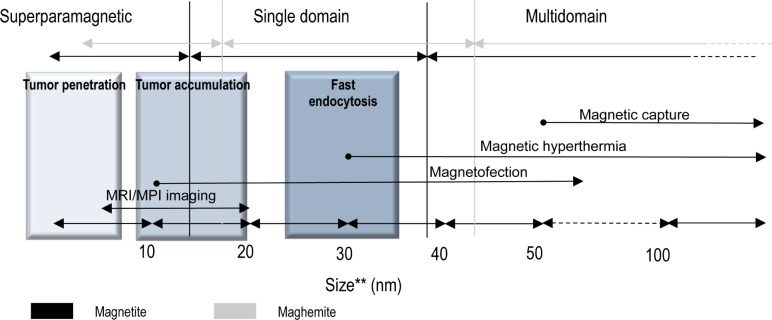
Schematic representation of the theoretical magnetic regimes (superparamagnetic, single domain, multidomain) expected for both magnetite and maghaemite, along with some relevant applications as a function of the particle size. (*) Magnetofection is a trademark of Christian Bergemann and Dr Christian Plank. (**) Refers to uncoated, single nanoparticles. The size ranges represented are approximate and comprise the most common cases.

On the biological side, the shape of an NP determines the extent to which the NP will interact with membrane receptors and hence the degree of internalization. Shi *et al.* modelled the particle–cell interaction by considering the diffusion and absorption of rod-like (cylindrical) and spherical NPs through a partially absorbing spherical cell [15]. For a fixed set of physical parameters of the particles, cell and medium, numerical results show that for each NP size there is a specific aspect ratio value for which the absorption rate is maximum (red region in Figure 2A), demonstrating the joint relevance of the size and shape of NPs to their internalization. Strange as it may seem, the outcomes of this work also suggest that the optimum particle size and shape for endocytosis do not depend noticeably on either the particular NP's absorption mechanism or the viscosity of the medium. Subsequent models based on similar grounds coincide in more general aspects. Noticeably, in particles with increasing aspect ratios, endocytosis becomes energetically favourable with decreasing particle sizes (Figure 2B). Particle orientation adds to the intricate influence of particle shape. Recent theoretical calculations based on membrane mechanics show how possible particle wrapping states—designated as *shallow* (Figure 2C) and *deep* (Figure 2D)—evolve, depending on the particle aspect ratio and edge curvature [16]. The proposed model predicts that particles with a high aspect ratio and round tips enter the cell membrane with their long edge parallel to the membrane, whereas those with low aspect ratio and sharp edges proceed tip-first. Despite its intrinsic limitations, this model provides the basis for making predictions of particle toxicity considering particles' shape and wrapping degree. Other studies on the interaction between other types of nanostructures, like nanosheets or nanotubes, and cell membranes have been reviewed elsewhere [17]. Although some of the results reported for these nanostructures sharply differ from those for NPs, many others are consonant; for instance, some aspects relating to the uptake mechanism of nanotubes and elongated NPs with variable edge curvature [17][18]. Finally, the circulation and the eventual *in vivo* fate of IONPs inside the body are also determined by particle shape to a great extent [19].

### 2.3 Surface charge density

An additional degree of complexity linked to the biological activity of NPs is constituted by their effective superficial charge, which determines the type of active or passive functionalization through specific molecules and the relevant particle–cell interaction mechanisms, which is revisited in the following sections. In this regard, the magnetic properties of NPs would remain virtually unaffected by their surface charge, even though the coating used to tune the surface charge or the affinity for a particular target may introduce sizeable modifications, as discussed later. As a first approximation, bare IONPs possess a surface charge strongly influenced by the environment, involving the pH and the ionic strength of the medium. The point of zero charge (PZC), that is, the pH values for which the surface charge density is zero, for bare IONPs may span several pH units depending on the type and concentration of charged species in the medium. For magnetite, it has been reported to range between 3.9 and 9.9 [20], and for maghaemite the reported range is around 3.3–7.5 depending on the reaction conditions [21]. Besides the PZC, another surface charge parameter of interest is the zeta-potential (ζ) [22][23], whose sign and value give an idea of the colloidal stability of NP suspensions rather than focusing exclusively on the isolated particles. It is commonly accepted that a stable colloidal suspension of IONPs has approximately ζ < −30 mV or ζ > +30 mV. Nevertheless, bare IONPs as such are of very limited interest for biomedical applications, due to their non-specificity, tendency to aggregation and short circulation times, as well as their toxicity, since they may induce vacuole formation and other cell damage leading to cell death [24]. In order to manipulate the surface charge, improve their biocompatibility and/or add a specific functionality (see section 2), IONPs can be coated with a large number of molecules by virtue of their relatively wide PZC range discussed above.

The surface charge of both bare and coated IONPs influences the binding to cell membranes as the limiting step in their internalization process [25], irrespective of the shape. It is rather the sign of the charge that determines the internalization mechanism [26]. Membrane interaction can occur regardless of the charge of NPs, but it is well known that positively- or negatively-charged IONPs are internalized more rapidly than neutral ones. For instance, *in vitro* studies with L929 fibroblasts and Saos-2 osteoblasts reveal how the uptake of negatively-charged SPIONs (coated with dimercaptosuccinic acid, DMSA) and positively-charged SPIONs (coated with (3-aminopropyl) triethoxysilane, APS) is far superior to that of neutral SPIONs (coated with dextran) [27]. This charge-mediated internalization enhancement may lead to cell damage for concentrations of DMSA and APS-coated SPIONs above 0.10 mg/mL in L929 fibroblasts and 0.20 mg/mL in Saos-2 osteoblasts. Mahmoudi *et al* have found that positively-charged SPIONs promote amyloid-β protein fibrillation at significantly lower particle concentrations than neutral or negatively-charged SPIONs [28].

A good example of how surface charge may affect the fate of similar IONPs formulations inside mammalians arises from the comparison of ferumoxides (ζ = −32 mV), ferumoxytol (ζ = −49 mV) and ferumoxtran (ζ = −2 to 0 mV). The latter has a thicker dextran layer [29] to avoid opsonization (protein adsorption) typically seen in very small particles and increase its circulation time, but in exchange is internalized to a lesser extent due to its almost neutral ζ. On the other hand, ferumoxides and ferumoxytol do not adsorb proteins and do not readily become opsonized, but are removed from circulation by the reticuloendothelial (RES) system due to their negative ζ.

## 3. Functionalization of IONPs

Functionalization of IONPs with biomolecules is a subject of great interest for two aspects related to their clinical applications. First, IONPs can be used as nanocarriers for drug delivery to tackle drug resistance of cancer cells and to increase local drug concentrations. Second, IONPs can be functionalized with specific targeting agents in order to improve the selectivity of specific cells, such as cancer cells, and therefore improve the selectivity, reduce side effects and increase local concentrations of drugs and/or IONPs in the targeted tissue. All these strategies are involved in the development of new generations of functionalized and multifunctionalized NPs for biomedical applications that introduce selective targeting properties to such nanocarriers.

The effect of targeting is strongly related to the enhancement of cellular uptake. Indeed, targeting strategies involve the recognition and binding to membrane receptors overexpressed on targeted cells, which changes the cellular uptake pathway and its efficiency. Several functionalization and targeting strategies have been developed in parallel with the latest advances in the discovery of new biomarkers specific to the different types of cells considered (e.g., cancer cells, cancer stem cells, etc.).

Currently, no clinical trials are in progress concerning the use of functionalized IONPs in humans. However, almost all the scientific publications in the field mention the potential biomedical and clinical applications of functionalized IONPs considering the promising *in vitro* and *in vivo* results. Among the wide diversity of molecules that have been used for the functionalization of IONPs described hereafter, most are already accepted by the different drug regulation administrations or are now in late clinical trial phases. Similarly, the IONP-based formulations ferumoxytol and GastroMARK are also approved. Therefore, the combination of both IONPs and specific functionalities shows great potential in the near future for the generation of novel nanoformulations for clinical use.

**Figure 3.**
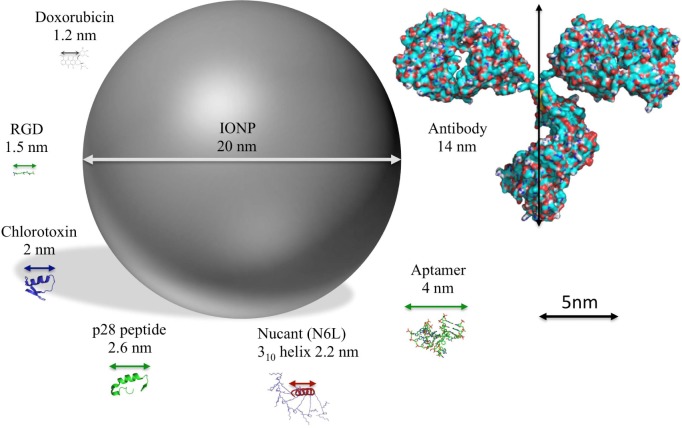
Functionalization of IONPs Schematic representation to scale of IONPs and the structure of different molecules used for their functionalization Structures represented: IONPs doxorubicin RGD peptide (PDB ID: 3VI4) chlorotoxin (PDB ID: 1CHL) azurin p28 peptide (PDB ID: 4AZU) Nucant (N6L) [52] aptamer (PDB ID 4HQU) and antibody (PDB ID: 1IGT).

Here, we present an overview of the different molecules that are currently used for the functionalization of IONPs, highlighting some relevant examples and their potential in clinics (Table 2 and Figure 3).

**Table 2.** Examples of biomolecules approved for clinical applications or already in clinical trials that are used in IONPs functionalization research studies

Type of biomolecule	Used name	Biological Target	Clinical trials status	Remarks and references of IONPs functionalization in research studies
Cell Penetrating Peptides (CPPs)	p28 (azurin fragment)	DNA binding domain of p53	p28 alone in phase I (NCT01975116 and NCT00914914)	No research studies with IONPs
Small Peptides	Arginine-Glycine-Aspartic acid (RGD)	Integrin receptor α_v_β_3_	Phase I/II for cancer diagnostic by positron emission tomography (NCT01806675, NCT00565721, NCT01492192, NCT01961583)	Montet et al. 2006 Xie et al. 2008 Nazli et al. 2012
	Chlorotoxin (CTX)	Binding affinity for gliomas and neuroectodermal tumors	Phase I for cancer imaging and safety study	Sun et al 2008
			(NCT00379132, NCT00733798, NCT00040573)	
	Nucant pseudopeptide (N6L)	Binding nucleolin and nucleophosmin	Phase I/IIa, Study to Assess Safety, Tolerability, Pharmacokinetics and Preliminary Efficacy on Advanced Solid Tumors (NCT01711398)	Latorre et al. 2014 (submitted) Destouches et al. 2011
Antibodies	Trastuzumab	Her2/neu receptor	Accepted by FDA and over the world (Herceptin as commercial formulation for breast cancer)	Hu et al. 2005
Aptamers	E10030	Anti-platelet-derived growth factor (anti PDGF-B)	Phase III for Age-Related Macular Degeneration (in complement with other anti-VEGF drug) NCT01940900	No research studies with IONPs
Folic Acid	Folate	Folate receptors	Several clinical studies of folic acid conjugated to anti-cancer drugs (NCT00485563, NCT00485563, NCT00291785, etc.)	Fan et al. 2011

### 3.1 Cell-penetrating peptides (CPPs)

Cell-penetrating peptides (CPPs), also known as protein transduction domains (PTDs) are up to 30-amino acid amphiphilic peptides that can be internalized by cells using mechanisms that require no energy and that can be receptor-mediated or not. Among the large variety of CPPs sequences, the presence of positively-charged amino acids and the amphiphilic character are the two common characteristics [30]. First used for intracellular delivery of macromolecules [31], CPPs are very interesting as drug and NPs intracellular delivery carriers. IONPs functionalized with different amounts of Tat peptide showed an exponential increase of cell uptake by increasing the number of CPPs grafted onto IONPs, concluding in a multivalent effect on the internalization [32]. The important parameters for the entrance of CPPs are positive charge and amphiphilic character. Martin *et al.* [33] and Cavalli *et al.* [34] studied the use of guanidine-based dendritic structures and synthetic peptides mimicking CPPs' properties for IONP functionalization. Both studies, with different cell lines, had comparable results to the ones observed with CPPs. The interest in using synthetic structures is due to their stability toward protease degradation. These examples show that the conjugation of IONPs with CPPs and analogue peptides with similar physical properties can be used to improve the cellular uptake of IONPs.

Additionally, newly identified tumour-homing CPPs have been discovered, expanding the use of CPPs for selective tumour targeting [35][36]. The only cell-penetrating peptide that has been tested in clinical trials is the so-called p28 peptide, which is derived from azurin protein and induces an increase of p53 and consequently cell death by apoptosis [37]. A completed phase I study (NCT00914914) and an ongoing phase I study (NCT01975116) have been carried out with the p28 peptide used as an anticancer drug for different types of tumours [38]. As far as we know, this p28 peptide has never been tested for IONP functionalization or for targeting strategies.

### 3.2 Antibodies and other proteins

Antibodies are complex protein molecules that bind to specific antigens with high affinity. Antibodies can be developed to bind to almost any given antigen, for example, specific receptors expressed on the surface of cells, and are therefore very useful tools to target disease biomarkers. Apart from their use as anticancer agents [39], antibodies have been used in IONP functionalization for their targeting properties toward cancer cells for a decade. As shown in the preliminary and encouraging study of Kang *et al.* in 2002 [40], IONPs can be functionalized with an antibody against E-selectin, an adhesion molecule induced in cancer cells. The final nanostructure was able to bind HUVEC cells specifically when the overexpression of E-selectin is previously activated.

Numerous research groups are interested in the functionalization of IONPs with trastuzumab, a commercial and approved antibody for breast cancer treatment [41], for the development of targeted therapeutic strategies. Most of the studies confirmed the selectivity of the antibody-functionalized IONPs toward cell lines overexpressing the Her2 receptor, depending on receptor expression level [42][43]. Vigor *et al.* showed similar results using another antibody that binds carcinoembryonic antigen (CEA), a receptor overexpressed in colorectal cancer cells [44]. The use of antibodies for intracellular uptake of IONPs is currently developing and several works have examined the cell line specifically targeting the enhancement of the uptake.

Additionally, other proteins are being used to functionalize IONPs and improve the cellular uptake and cell targeting. The example of transferrin-functionalized IONPs to target cancer's overexpression of transferring receptors has been recently published by Piraux *et al.* in 2013 [45].

### 3.3 Targeting peptides

Apart from macromolecules such as antibodies and CPPs, smaller peptides also interact with specific receptors in the cell membrane and increase the cellular uptake of the functionalized IONPs.

Arginine-glycine-aspartic acid, also known as RGD peptide, binds to the integrin receptor (α_v_β_3_) which is overexpressed in several cancer cell lines and is commonly used as a targeting agent. Several works show that the functionalization of IONPs with RGD peptide (in linear or cyclic conformation) induces a significant increase in the cell uptake, compared to bare IONPs, specifically for α_v_β_3_ positive cell lines as in some cancer cell lines (BT-20 and HeLa) and no significant changes in α_v_β_3_ negative cells (9L, MCF-7, or U87MG cells) [46][47][48]. Similarly, chlorotoxin (CTX) is a 36-amino acid peptide that has a high selectivity and binding affinity for gliomas and neuroectodermal tumours [49]. Sun *et al.* showed a tenfold increase in the cellular uptake of CTX-functionalized IONPs compared to bare IONPs in 9L cells [50]. Nucant pseudopeptide (N6L) is a 4-kDa pseudopeptide currently in phase II clinical trials (NCT01711398), while the MultiFun European project (EU-FP7 no. 262943) is currently working on the multifunctionalization of IONPs with N6L and anticancer drugs. The significance of this pseudopeptide is that it acts as a targeting agent through its specific binding to nucleolin and nucleophosmin (overexpressed in several cancers) and also as a therapeutic agent [51][52]. The first results obtained showed the targeted uptake of N6L covalently-functionalized IONPs and the controlled release of N6L in a reducing medium which mimics the intracellular environment

### 3.4. Aptamers

Aptamers are single-stranded oligonucleotides that bind to their specific targets with high efficiency. Aptamers have been used for the targeting and specific cellular uptake of IONPs, as they present several advantages, such as their easy and reproducible synthesis, their good stability and their lack of immune response. Chen *et al.*, in 2011, developed PEG-IONPs functionalized with doxorubicin and sg8c aptamer. Sg8c binds to the cell membrane receptor protein tyrosine kinase 7 (PTK7). The study showed a clear specificity of sg8c-functionalized IONPs for PTK7 positive cells compared to PTK7 negative cells in correlation with the cytotoxicity of released doxorubicin under high pH of lysosome medium, which also showed a receptor-mediated entrance [53].

### 3.5 Carbohydrates

Functionalization of IONPs with carbohydrates can improve cellular uptake by two different strategies: (1) providing higher hydrophilicity and biocompatibility and thus increasing lifetime in the bloodstream; and (2) the interaction with specific sugar receptors on the membrane of certain cell lines, including cancer cells [54]. Moros *et al*. studied the uptake of IONPs functionalized with monosaccharides (glucose or galactose) compared to IONPs functionalized with PEG and showed that the type of carbohydrate affects the rate and the mechanism of internalization [55]. In 2011, Valero *et al*. also showed interesting results using IONPs encapsulated within apoferritin (apomaghemite) and then functionalized with two carbohydrates, N-acetyl-D-glucosamine and D-mannose. First, they found similar behaviour to an MRI contrast agent such as the commercial agent Endorem. Finally, they also observed that both carbohydrate-functionalized apomaghemite NPs retained the carbohydrate lectin recognition activity [56].

### 3.6 Folic acid

Folic acid, also known as Vitamin B9, is a natural product with an important role in DNA and RNA synthesis. Over 20 years, folate receptors have been shown to be overexpressed on several cancer cell lines and can therefore be used for targeting cancer cells and activated macrophages [57][58]. Folic acid has already been approved for several clinical applications. Clinical trials are in progress for drug-folate conjugate use in cancer diagnosis (NCT00003763) and therapy (NCT00291785, NCT00485563, NCT00441870). Folic acid has been widely used for 15 years in the development of NP targeting strategies for biomedical applications, including IONPs. We highlight the work of Fan *et al.* in 2011, where a significant enhancement of cellular uptake of the folic acid-functionalized IONPs is achieved compared to bare IONPs, depending on the receptor expression levels of the cell lines used. Promising results of MRI *in vivo* highlight the potential of folic acid-functionalized IONPs for clinical applications [59].

### 3.7 Drugs

The functionalization of IONPs with drugs for their application as nanocarriers is a wide and much studied topic [60]. Indeed, the major portion of the drugs used in combination with IONPs at the research level are already available in clinics, such as doxorubicin, gemcitabine and SN38, and are also involved in several clinical trials of drug-targeting agent conjugates. Currently, there are some promising examples of doxorubicin conjugated with antibodies now in clinical trials (NCT00051584), and the use of liposomal formulations for drug delivery (NCT01227941).

The multifunctionalization of IONPs by targeting agents and drugs, and its acceptance in clinical processes, might be a breakthrough in the development of specific therapies. The presence of targeting moieties such as those discussed above will increase the localization of IONPs in the diseased tissue, therefore augmenting drug delivery and local drug concentration. Thus, research is currently making efforts towards the multifunctionalization of IONPs. Different methodologies need to be developed to ensure controlled multifunctionalization, and preserve the stability and biological activities of active molecules attached to the particles. Additionally, with respect to the clinical application of these nanostructures, functionalization efforts are required to increase the blood half-life of the IONPs, minimizing off-target accumulation and immunological response.

## 4. Toxicological profiles and potential interference with biological functions of cells

**Figure 4.**
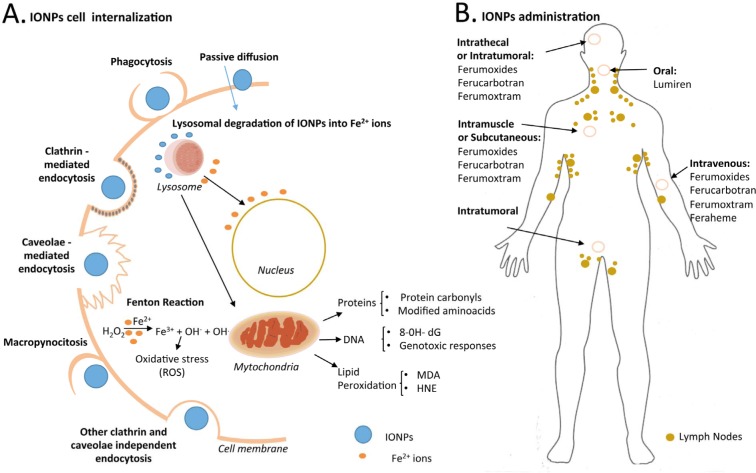
A. Different internalization pathways of IONPs in mammalian cells. Possible mechanisms of uptake including macropinocytosis, caveolae- and clathrin-mediated endocytosis, phagocytosis, passive diffusion and other endocytosis pathways. After internalization, IONPs can produce cytotoxicity effects via a Fenton reaction. Hydroxyl radicals generated could damage DNA, proteins or lipids (8-OH-dG = 8 hydroxydeoxyguanosine, MDA = malondialdehyde, HNE = 4-hydroxy-2-nonenal), triggering genotoxicity. B. IONP administration in the human body, such as intrathecal, intratumoural, intravenous and intramuscular or subcutaneous methods.

The above-mentioned parameters have a tremendous influence on cell internalization as well as *in vivo* behaviour, and hence in the administration procedure of choice for clinical applications [61]. Mammalian cells internalize IONPs mainly through endocytosis mechanisms. However, the specific pathway depends on the cell type and physicochemical features of the IONPs. Most cells use different molecular pathways of pinocytosis, whereas cells from the reticuloendothelial system (RES) preferentially internalize IONPs by phagocytosis in a process that begins with the opsonization of the NPs in the bloodstream, followed by binding of the opsonized NPs to the cell surface and final ingestion [62] (Figure 4A). The IONPs approved for clinical use are not functionalized, but they display a variety of sizes that influence their administration and distribution within the body. SPIONs and USPION accumulate in the lungs, liver, spleen, lymph nodes, bone marrow vessels and capillaries, while VSPIONs also accumulate in the kidneys (Figure 4B). Once in the body, the iron of the NP core is stored in the red blood cells and, like the endogenous iron, it is progressively eliminated via faeces, while the coating (mostly dextran-derived compounds) degrades and is eliminated by the kidneys. However, the IONPs interact with different biological systems in the body and may have adverse effects. Evaluating the toxicological effects of IONPs both *in vitro* and *in vivo* is crucial for the development of IONP-derived applications in medicine.

### 4.1 Effects on cellular physiology

#### 4.1.1 Mitochondrial stress

One factor that can contribute to nanotoxicity is the size of the NPs. Smaller NPs have a greater reactive surface area than larger ones, are more chemically reactive, and produce greater numbers of reactive oxygen species (ROS) that include free radicals [63]. SPIONs also can induce (geno-)toxicity via generation of ROS. SPIONs are presumably degraded into iron ions within the lysosomes by hydrolysing enzymes effective at low pH [64]. This “free iron” can potentially cross the nuclear or mitochondrial membrane and, in the latter case, the free iron in the form of ferrous ions (Fe^2+^) can react with hydrogen peroxide and oxygen produced by the mitochondria to produce highly reactive hydroxyl radicals and ferric ions (Fe^3+^) via the Fenton reaction (Figure 3a). Therefore, hydroxyl radicals generated by the free iron could damage DNA, proteins, polysaccharides and lipids *in vivo* [65]. The cytotoxicity was also found to be dependent on various factors, such as type of surface coating or its breakdown products, chemical composition of cell medium, oxidation state of iron in SPION and protein–SPION interaction [24][66][67]. For example in IONPs, magnetite (Fe_3_O_4_) and maghaemite (Fe_2_O_3_) can show different cellular responses because of their ability to undergo oxidation/reduction reactions. In fact, magnetite has been shown to cause higher levels of oxidative DNA lesions in the A549 human lung epithelial cell line in the absence of decreased cell viability as compared to maghaemite, owing to its potential to undergo oxidation. It is hypothesized that the toxicity can, however, be decreased by coating magnetite NPs, resulting in fewer oxidative sites that are less reactive, thereby producing less DNA damage [66].

#### 4.1.2 Effects on actin cytoskeleton architecture

Intracellular accumulation of NPs can have profound effects on cell morphology. High levels of internalized particles can provoke cellular stress, inducing changes in the architecture of the actin cytoskeleton. An altered actin network displaying actin stress fibres can lead to a reduced proliferative capacity and cell spreading. It can also influence the migration and differentiation of stem cells.

In the case of IONPs, it has been reported to interfere with actin and tubulin structures, inducing cell retraction, rounding and deposition of massive dense filament matters adjacent to the nucleus and vacuoles in the cytoplasm [68].

### 4.2 Factors that can contribute to non-toxicity

Size is clearly a key factor in determining the potential toxicity of a particle. However, it is not the only important factor. Other properties of nanomaterials that influence toxicity include chemical composition, shape, surface structure, surface charge, aggregation and solubility [63]. A study investigating the effect of different surface coatings on cell behaviour and morphology has shown that dextranmagnetite (Fe_3_O_4_) NPs result in cell death and reduced proliferation similar to that caused by uncoated IONPs [24]. Other authors have reported the formation of gas vesicles after exposure to the uncoated NPs, resulting in altered protein functions and changes in ionic equilibrium within the cells, which also promotes toxicity [69].

### 4.3 In vitro and in vivo toxicity of nanoparticles in 2D vs. 3D cell culture

Common 2D cell cultures do not adequately represent the functions of 3D tissues that have extensive cell–cell and cell–matrix interactions, as well as markedly different diffusion/transport conditions. Hence, testing cytotoxicity in 2D cultures may not accurately reflect the actual toxicity of NPs and other nanostructures in the body [70]. For instance, recent studies on toxicity testing of magnetic NPs, using *in vitro* 2D cell culture, demonstrated high cytotoxic effects. However, when they were tested in animal models, no adverse effects were observed [71]. Therefore, *in vitro* 3D cell culture models have been introduced to bridge the gap between *in vitro* 2D cell culture and *in vivo* models. Some authors report that bare IONPs were more toxic at lower concentrations in 3D culture, compared with 2D culture cells. This may be due to the increased contact area between NPs and cells in 3D culture [72].

When IONPs enter the body, absorption can occur through interaction with biological components such as proteins and cells; afterwards, they can distribute into various organs where they may remain in the same nanostructure or become metabolized [73]. A systematic and thorough quantitative analysis of the pharmacokinetics (i.e., absorption, distribution, metabolism and excretion) of IONPs can lead to improvements in design of biocompatible IONPs, a better understanding of NPs' non-specificity toward tissues and cell types, and assessments of basic distribution and clearance that serve as the basis to understand their activity and potential toxicity [74].

The surface of IONPs is rapidly covered by selective sets of blood plasma proteins after injection. Small IONPs (< 10 nm) are usually rapidly removed through extravasation and renal clearance, whereas large IONPs (> 200 nm) are sequestered by the spleen via mechanical filtration [75]. The typical final biodistribution of IONPs is 80–90 in liver, 5–8% in spleen and 1–2% in bone marrow, due to the high number of macrophages contained in these organs [61]. IONPs were also found to be distributed in the brain, liver, spleen and lungs after their inhalation, demonstrating their ability to cross the blood–brain barrier [76] (Figure 4A).

## 5. Current clinical applications

In December 1996, the United States Food and Drug Administration (FDA) approved GastroMARK (AMAG Pharmaceuticals), an aqueous suspension of silicone coated, superparamagnetic iron oxide NPs, intended for oral administration, as a magnetic resonance (MR) imaging contrast medium to enhance the delineation of the bowel to distinguish it from organs and tissues that are adjacent to it in the upper regions of the gastrointestinal tract (www.fda.gov). The approval of GastroMARK, together with a number of preclinical studies supporting the potential use of SPIONs in different medical applications, contributed significantly to expanding clinical research in nanomedicine. A number of new SPION-derived products were developed, some of which were also approved by the FDA and the European Commission (EC) for clinical use. Additionally, the increasing number of clinical trials using these new NPs expanded their potential medical applications. Sixteen years later, just two SPION-derived products are approved by the FDA and commercially available: GastroMARK and ferumoxytol (Table 3). In the following paragraphs, we will describe the current clinical applications of SPION-derived products, as well as the clinical trials testing them for promising applications such as cell tracking and hyperthermia.

**Table 3.** Iron oxide nanoparticles (ION) for clinical applications: the table displays the chronology of ION approved by the Food and Drug Administration (FDA) and/or the European Commission (EC), discontinued and/or production abandoned. Sinerem's Authorization Application was withdrawn by Guerbet (European partner of AMAG Pharma) in 2007. Ferumoxide was discontinued by AMAG Pharma in 2008 and the production of Retrovist was abandoned in 2009.

Date	Events	Indication	Size	Adm.
1996	Lumirem (Gastromark (US)): approved by FDA	Contrast agent for MRI for the gastrointestinal tract	50 nm	Oral
1996	Ferumoxides (Emdorem (EU) or Feridex 1.V.): approved by the FDA	Contrast agent for MRI of liver lesions	120–180 nm	Injectable solution
2001	Ferucarbotran (Resovist or (Cliavist(EU)): approved for Europena Market	Contrast-enhanced MRI of the liver	45–60 nm	Injectable solution
2005	Ferumoxtram (Sinerem (EU) or (Combidex (US)): AMAG Pharma received an approvable letter from the FDA	Detection and characterization of metastatic lymph nodes in patients with pelvic cancer	10–40 nm	Injectable solution
2006	Sinerem: submitted European Marketing Authorization Application	Detection and characterization of metastatic lymph nodes in patients with pelvic cancer	10–40 nm	Injectable solution
2009	Feraheme (Ferumoxytol): approbed by the FDA	Iron replacement therapy for the treatment of iron deficiency anemia in adult patients with chronic kidney disease.	20–50 nm	intravenous
2012	Feraheme (Ferumoxytol): approbed by the EC	Iron replacement therapy for the treatment of iron deficiency anemia in adult patients with chronic kidney disease.	20–50 nm	intravenous

### 5.1 Imaging

**Figure 5.**
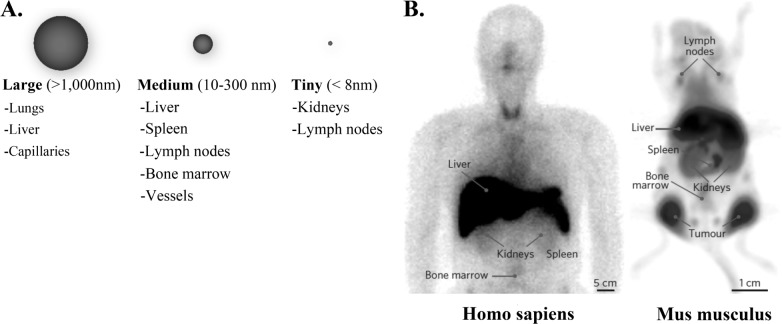
Organ distribution of systemically injected nanoparticles. a. Common organ distribution is shown as a function of particle size. b. Example of the ^99m^Tc-labeled graft copolymer used in a human patient and ^89^Zr-labeled cross-linked dextran nanoparticles used in a mouse model. This figure was reproduced with permission from *Nature Materials* [126].

Clinic imaging technologies rely on the use in MRI, optical imaging/MRI and MRI/PEP. SPIONs function as a negative contrast agent, decreasing T2 signals and thus the signal intensity. They cause a magnetic field gradient that affects the surrounding protons of water molecules, disrupting the homogeneity of the magnetic field, which can be observed by MRI [77]. Additionally, SPIONs display lymphotropic properties, as phagocytes of the RES internalize them, causing local changes in magnetic properties. These features make the SPIONs an excellent alternative to complement current contrast agents in MRI, providing higher accuracy in some conditions such as autoimmune diseases, cardiovascular and cerebrovascular pathologies, and cancer (Table 4). Additionally, the low toxicity both *in vitro* and *in vivo*, and their feasibility in enhancing the contrast of cellular targets in MRI, make then suitable for *in vivo* tracking of transplanted cells (Figure 5) [78].

**Table 4.** ClinicalTrials.gov search with the number of results and reference ID. Search terms: Superparamagnetic OR Iron Oxide OR SPIO OR USPIO OR VSPIO.

ClinicalTrials.gov	Imaging					Therapy		
Indication	Lymph nodes	Head and neck cancer	Cardiovascular and Cerebrovascular diseases	Autoimmune diseases	Cell tracking	Delivery	Anemia	Hyperthermia
**April 2014 (53)** **Clinical Trial Identificator**	10 NCT01815333 NTC00147238 NTC00920023 NCT01927887 NCT00188695 NCT00147238 NCT01296139 NCT00243594 NCT00416455 NCT00107484	7 NCT01895829 NCT0660543 NCT00769093 NCT01663090 NCT00978562 NCT00659126 NCT00659334	7 NCT01127113 NCT01323296 NCT01995799 NCT01674257 NCT01749280 NCT00794092 NCT02084303	4 NCT01973517 NCT00585936 NCT02006108 NCT01243320	6 NCT00972946 NCT01169935 NCT01127113 NCT00660543 NCT00243594 NCT00781872	3 NCT01270139 NCT01436123 NCT01927887	14 NCT01052779 NCT01114217 NCT01114139 NCT00114204 NCT01374919 NCT01264679 NCT01227616 NCT01155388 NCT01942460 NCT00233597 NCT00255437 NCT01148745 NCT00255450 NCT01950247	2 NCT02033447 DRKS00005476

#### 5.1.1 Diagnostic tools

##### 5.1.1.1 Diagnosis in immunological diseases

Autoimmune disease and inflammation processes are characterized by the activation of the endothelium that eventually allows for the extravasation of macrophages from the bloodstream into the inflamed tissue. SPIONs significantly enhance the signal of these events when analysed by MRI, as they are quickly captured by macrophages. In the last few years, a number of authors have taken advantage of this observation and carried out different clinical assays to test the value of SPIONs as a negative contrast in a variety of autoimmune and inflammatory conditions. Oral USPION (ferumoxytol) improved the detection of Crohn's disease [79] and the prognosis of active ulcerative colitis [80][81]. Using ferumoxtran, a significant improvement was observed in a small cohort of Type 1A diabetes patients (NCT00585936) in the diagnosis of pancreatic islet inflammation when compared to T1-gadolinium [82]. In patients with multiple sclerosis, USPION-based MRI showed that iron oxide NPs labelled larger and better defined areas, allowing the detection of what has been referred to as normal-appearing white matter that is histologically affected (NCT01973517) [83][84]. These outstanding results have encouraged physicians and researchers to continue testing the SPION-based MRI in both the previous and new immunological and inflammatory conditions. At least two more clinical trials were running at the time of writing this review (Table 4).

##### 5.1.1.2 Diagnosis in cardiovascular and cerebrovascular pathologies

The use of SPIONs for T2 and T2-weighted cardiovascular magnetic resonance imaging (CMR) has been successfully validated to detect myocardial oedema and myocardial haemorrhage [85][86]. In a more recent trial, Vilmaz *et al*. demonstrated that ferumoxytol significantly improved the signal in the myocardial infarction area when compared with gadolinium-based contrast images. This improvement was due to the accumulation of USPIONs in infiltrating macrophages [87]. Similarly, Sigovan *et al*. demonstrated that ferumoxytol-enhanced MR angiography rendered superior image quality compared with non-enhanced time-of-flight MR when studying upper extremity autogenous fistulas in patients with renal failure. The use of ferumoxytol reduced flow artefacts while simultaneously raising lumen depiction scores, thus obtaining a more homogeneous luminal signal enhancement [88]. The ability to detect macrophage activity of plaque inflammation by MRI has also supported the use of SPION-based MRI of carotid atherosclerosis to predict cerebrovascular and cardiovascular morbidity and mortality. The author found an interesting association between USPIO-defined plaque inflammation and the development of vascular events, although the association was not significant due, in part, to the relatively low baseline risk of vascular event of the patient included in the clinical trial [89]. However, the overall data available from different trials aim to leverage the SPION-based MRI to assess the diagnosis of cardiovascular and cerebrovascular pathology in the next year. Thus it will be very important to pay special attention to the results derived from the ongoing clinical trials (Table 4).

##### 5.1.1.3 Diagnosis in cancer

In healthy liver, spleen and lymph node tissue, IONPs decrease the MR signal intensity as phagocytic cells uptake the nanoparticles, while malignant tissue generally fails to uptake IONPs and appears bright relative to the surrounding tissue [90][91]. This observation emphasizes the IONP-enhanced MRI as an extremely promising imaging technique for the preoperative detection of metastatic disease in lymph nodes. In 2003, Harisinghani *et al*. reported that high-resolution MRI with IONPs (Sinerem) allowed the detection of small and otherwise undetectable lymph-node metastases in patients with prostate cancer, demonstrating the sensitivity and specificity of the technique [92]. From this point of departure, a number of clinical trials have successfully tested the IONP-enhanced MRI to detect sentinel lymph nodes in prostate cancer [93][94], as well as other types of cancers like bladder [95], breast (Endorem [96] and Resovist [97][98]) and renal cell (Sinerem) [99], among others (Table 4).

#### 5.1.2 Cell tracking in cell transplantation-mediated therapies

Cell therapy relies on the delivery of cells to the target site. Monitoring and tracking these cells to ensure tissue delivery and engraftment become a key issue in stabilizing clinical safety and therapeutic efficacy. In this sense, detection by MRI of IONP-labelled cells may be one of the most promising approaches for a short-term evaluation [100]. Although the FDA and EC have not yet approved their use for *in vivo* cell tracking, a few clinical trials have evaluated their feasibility and safety in different contexts (NCT00972946, NCT01169935 and NCT01127113). To evaluate the feasibility, safety and immunological effects of intrathecal and intravenous administration of autologous mesenchymal stem cells in patients with multiple sclerosis (MS) and amyotrophic lateral sclerosis, Karussis *et al*. designed and carried out a clinical trial in which MSC were magnetically labelled with ferumoxides. The MSC were incubated with ferumoxides in the presence of poly-L-lysine for 24 to 48 hours prior to transplantation. MRI examination of the brain and spinal cord was performed at 24 and 48 hours, and later at one to three months. The image revealed a possible migration of such cells to the meninges of the spinal cord and nerve roots, and to the spinal cord parenchyma, although they could not rule out the possibility that macrophages had phagocytized the iron oxide magnetic NPs and migrated to the inflammatory MS lesions [101]. In a different work, Richards *et al*. successfully SPION-labelled human peripheral blood mononuclear cells (PBMC) and performed intramuscular and intravenous administration of labelled cells in healthy volunteers without remarkable side effects. After administration of labelled cells, the signal intensity was reduced in the liver and spleen, reflecting dose-dependent accumulation of SPION-labelled cells. This work demonstrated that labelled cells can be visualized *in vivo* at clinically relevant field strengths after local or systemic administration, and that SPION-labelled cells can be tracked to a site of focal inflammation in humans. More importantly, however, they demonstrated that intravenous administration of SPION-labelled cells in humans is safe [78].

Cancer immunotherapy approaches can also benefit from SPION labelling technology. Dendritic cell (DC) vaccines have been used to induce tumour-specific cytotoxic T cells. To be successful, injected DCs need to migrate to the lymph nodes (LNs) where they can stimulate effector T cells. DCs have previously been labelled with radionuclides for scintigraphic imaging, which is the only clinical cellular imaging modality approved by the FDA [102][103]. deVris *et al*. investigated the biodistribution of SPION-labelled DCs applied as cancer vaccines in melanoma patients using MRI, and demonstrated that MRI allowed assessment of the accuracy of DC delivery and of inter- and intranodal cell migration patterns [104].

### 5.2 Therapy

#### 5.2.1 Delivery

Iron oxide NPs have been successfully used as a carrier for bioactive molecules in a number of conditions both *in vitro* and in animal models [105]. In the last few years, SPIONs have also been demonstrated to be useful in clinical settings (NCT01270139, NCT01436123 and NCT01927887). In 2009 Provenzano *et al*. reported that rapid intravenous injection of ferumoxytol led to significantly greater haemoglobin increases compared with oral iron, and was also well tolerated in all patients [106]. The same year, ferumoxytol was approved by the FDA, and later in 2012 by the EC, as an iron replacement therapy indicated for the treatment of iron deficiency anaemia in adult patients with chronic kidney disease. Since then, a number of clinical trials have extended the use of ferumoxytol as intravenous iron delivery/therapy to other iron deficiency conditions. Ferumoxytol was effective and well tolerated in adult patients with iron deficiency anaemia in whom oral iron was ineffective or could not be used [107][108], and showed comparable efficacy and adverse event rates to the first choice, iron sucrose [109], even in paediatric patients [110].

#### 5.2.2 Hyperthermia

Thermotherapy of solid tumours is one of the most promising applications of SPIONs, either alone or in combination with adjuvant treatments, such as chemotherapy or radiation. The principle of applying IONPs to hyperthermia therapy involves the administration of an IONPs fluid within the tumour, followed by the application of an alternating magnetic field. These nanoparticles achieve high temperatures, promoting warming of the region. Temperature elevation in the range of 41–46 °C can induce several effects at both cellular and tissue levels [111]. Cellular hyperthermia induces heat stress, resulting in high level of heat-shock protein expression, protein denaturation and folding and apoptosis. At the tissue level, temperature elevation induces changes in pH, perfusion and oxygenation of the tumour microenvironment [112][113], while higher temperature (hyperthermia) or longer treatment time may lead to necrosis.

Clinical studies for the application of thermotherapy using IONPs in humans were initiated in 2007 on prostate cancer and glioblastoma patients (NCT02033447 and DRKS00005476). Johannsen *et al*. injected the IONPs fluid transperineally into the prostate of ten patients who received six thermal therapies of 60 minutes duration at weekly intervals. They reached maximum temperatures of 55 °C in the prostate with a median temperature of 40.7 °C within the tumour and 40.2 °C in peritumoural zones. With this assay they demonstrated the feasibility of thermotherapy [114]. Afterwards, they carried out a different clinical assay demonstrating that interstitial heating using IONPs was feasible and well tolerated in patients with locally recurrent prostate cancer, and the deposition of nanoparticles in the prostate was highly durable [52].

In parallel, Maier-Hauff *et al*. evaluated the feasibility and tolerability of thermotherapy in patients with recurrent glioblastoma multiforme. In this trial, patients received four to 10 thermotherapy treatments, reaching a median intratumoural temperature of 44.6 °C. The authors observed that thermotherapy using IONPs was tolerated well by all patients with minor or no side effects [115]. A few years later, they carried out another clinical trial and observed that the median overall survival following diagnosis of first tumour recurrence was 13.4 months, which was an improvement compared to the 6.2 months in other reference studies in the field. Additionally, the median of overall survival after primary tumour diagnosis was 23.2 months, compared with just 14.6 in the reference groups [116].

More recently, Matsumine *et al*. reported the utilization of hyperthermia in patients with metastatic bone tumours. The authors carried out an initial surgical intervention followed by the implantation of a mixture of “bare” magnetite NPs and calcium phosphate cement, which is a biocompatible bone substitute. The patients were treated for 15 minutes every two days starting from the eighth day after surgery. Thirty-two percent of lesions were reduced and presented visible bone formation, 64% showed no progressive lesions for more than three months and just 4% presented a poor response to the treatment, demonstrating safety and effectiveness [117].

Although these results are promising, the development of therapies based on magnetic hyperthermia is still in its very early stages. In the next few years, many biological and technical advances need to be achieved before this new therapy becomes part of the standard of care for some cancers. Questions such as how to reach homogenous distribution of IONPs and therapeutic temperature, and how to minimize peritumoural tissue damage, need to be addressed. In this regard, a new phase 0 clinical trial to investigate the magnetic nanoparticle thermo-ablation-retention and maintenance in the prostate is being performed by the University College London Hospitals (NCT 02033447). In this trial, the authors will test whether the magnetic nanoparticles actually stay where they are injected or move to sensitive structures around the prostate, which may lead to undesirable side effects. Finally, MagForce is conducting a new open-label, randomized clinical trial employing magnetic hyperthermia to treat glioblastoma multiforme patients (DRKS00005476). The purpose of this trial, expected to enrol up to 285 patients, is to test the feasibility and safety of using magnetic hyperthermia as a stand-alone therapy and in combination with radiotherapy using the commercial IONPs NanoTherm®. The results will help magnetic hyperthermia advance in the clinical setting.

## 6. Perspectives

Bare IONPs are currently employed in an increasing number of clinical trials. In the next decade, we will see them settle into clinical practice for imaging-based diagnosis for a wide range of diseases. One of the main entries of IONPs into the scene of medical application will surely come from their functionalization possibilities. These will provide them with the capacity to target specific cells within the body, and hence develop specific therapies and diagnostic tools. The main advantages of using nanocarriers for specific drug delivery are the reduction of side effects, potential drug resistance and increased drug payload. As unique targeted nanocarriers can deliver hundreds of small drug molecules, clinical practices shall be adapted to incorporate these new strategies. They should include treatment protocols personalized by choosing appropriate formulations with defined targeting moieties and drug loads. The multifunctionalization of IONPs opens the door to multimodal therapeutic approaches by combining the dual effects of magnetic hyperthermia and chemotherapy, using multiple drugs in the same IONP formulation. Additionally, functionalization of IONPs will allow researchers to tune and optimize IONPs' biocompatibility, blood half-life and immunogenicity, key parameters required to successfully bring these innovative nanotechnology-based tools from research laboratories to clinics. Overall, the multifunctionalization of IONPs is a critical step. It is most likely that it will be broadly implemented in the near future for new nanoformulations for both diagnosis and therapy.

In the particular case of IONPs for magnetic hyperthermia, a number of fundamental and practical aspects still require more research. One of these concerns the magnetic and mechanical behaviour of IONPs inside cells: specifically, we refer to lysosomes under dynamic external fields. Although a few theoretical studies addressing this matter have appeared [118], we require specific experimental approaches to confirm their predictions. Additionally, more work needs to be done to determine the actual heating mechanisms under common frequency and field amplitude conditions, and to evaluate the involvement of any other physical phenomena. In this sense, the results from a very recent publication exploring possible damage to the lysosomal membrane by a remotely induced torque over IONPs are particularly encouraging [119]. The role of phonon-mediated heating and its coupling to those biomolecules in the surroundings, or directly linked to IONPs, is now receiving more attention [120]. These studies will enable us to discern whether or not a sizeable macroscopic temperature change is required to have a therapeutic effect. If not, temperature changes on the micro- or nano-scale could be enough to induce cell death or, at least, to sensitize cancer cells to other treatments (chemo/radiotherapy). Finally, the possibility of having magnetic hyperthermia in an MRI machine has been proposed [121–122], but no clear steps have been taken to further develop this appealing idea.
